# Competitive Sequestration of miR-1183 by lncRNA DDX11-AS1 Drives Gliomagenesis through E2F7 Activation

**DOI:** 10.32604/or.2025.065380

**Published:** 2025-09-26

**Authors:** Jianwei Wang, Xinzhi Yang, Lvbiao Lin, Jianbo Yu, Jie Mao

**Affiliations:** 1 Department of Neurosurgery, Shenzhen Hospital, Southern Medical University, Shenzhen, 518100, China; 2 The Third School of Clinical Medicine, Southern Medical University, Shenzhen, 518100, China; 3 Department of Neurosurgery, Shenzhen Yantian District People’s Hospital, Shenzhen, 518100, China; 4 Department of Neurosurgery, Longgang Central Hospital of Shenzhen, Shenzhen, 518100, China; 5 Department of Pathology, Longgang Central Hospital of Shenzhen, Shenzhen, 518100, China

**Keywords:** Glioma, DDX11 antisense RNA 1, miR-1183, E2F transcription factor 7, competitive endogenous RNA

## Abstract

**Objectives:**

Glioma, as the most lethal primary brain malignancy with poor prognosis, requires further elucidation on the functional role of long noncoding RNA (lncRNA) DDX11 antisense RNA 1 (DDX11-AS1) in its pathogenesis, despite its established oncogenic functions in other cancers. Therefore, this study sought to characterize the oncogenic role and molecular mechanism of DDX11-AS1 in glioma.

**Methods:**

DDX11-AS1 expression levels were analyzed in clinical surgical glioma specimens and publicly available datasets. The functional roles of DDX11-AS1 on glioma cell proliferation and migration were investigated using *in vitro* knockdown and overexpression assays. *In vivo* tumor growth was assessed using orthotopic glioma-bearing mouse models. To elucidate the regulatory axis involving DDX11-AS1, miR-1183, and E2F transcription factor 7 (E2F7), we performed competitive endogenous RNA (ceRNA) analysis and conducted functional rescue experiments via miR-1183 inhibition.

**Results:**

DDX11-AS1 expression was markedly upregulated in clinical glioma specimens. Functionally, DDX11-AS1 knockdown significantly suppressed glioma cell proliferation and migration *in vitro*, while its overexpression exacerbated these malignant phenotypes. Orthotopic glioma-bearing mouse models confirmed that DDX11-AS1 drives *in vivo* glioma tumor growth. Mechanistically, DDX11-AS1 functions as a ceRNA by competitively interacting with miR-1183. Critically, inhibition of miR-1183 rescued the suppressive effects of DDX11-AS1 knockdown on glioma tumorigenic phenotypes and restored E2F7 expression levels.

**Conclusions:**

This study demonstrates that lncRNA DDX11-AS1 promotes glioma progression by regulating the miR-1183/E2F7 axis, indicating a potential therapeutic target for glioma.

## 1 Introduction

Glioma comprises the predominant form of primary brain tumors in adults, and is associated with obstinately high morbidity and mortality rates. Glioblastoma, specifically WHO grade 4 glioma, is the most aggressive and lethal form. Despite combined aggressive therapy of surgery, chemotherapy and radiotherapy, the outcomes of patients with glioma remain suboptimal, with the mean overall survival less than 15 months [1,2]. Several critical genes, transcripts and signaling pathways are important tumorigenic factors contributing to the development of glioma, and biomarker discoveries have greatly improved the understanding and management of many types of cancers [3,4]. Therefore, a deeper investigation into the molecular mechanisms underlying glioma malignant progression could provide crucial insights into novel therapeutic targets and eventually improve prognosis.

More than 75% of the human genome is transcribed into long non-coding RNAs (lncRNAs) and microRNAs. lncRNAs are defined as a class of RNA molecules transcripts longer than 200 nucleotides without protein-coding capacity [5]. Recently, several studies have suggested that lncRNAs play crucial roles in glial development and astrocyte terminal differentiation in the developing central nervous system [6,7]. Reflecting their important role in genetic modulation processes, the dysregulation of lncRNAs has been implicated in the progression of human cancers [8–10]. DDX11 antisense RNA 1 (DDX11-AS1), which is located at human chromosome 12p11.21, has recently been recognized as a cancer-related lncRNA [11,12]. Increasing studies have shown that DDX11-AS1 may be involved in oncogenic regulation across diverse malignancies [13–15]. For instance, expression of lncRNA DDX11-AS1 significantly elevated in hepatocellular carcinoma patients compared with that in normal controls. DDX11-AS1 promoted the formation of hepatocellular carcinoma by suppressing the expression of large tumor suppressor kinase 2 [13,16]. Furthermore, lncRNA DDX11-AS1 was up-regulated in gastric cancer and served as an oncogene by sponging miR-873-5p and miR-326, respectively, eventually promoting gastric cancer progression [15,17,18]. Additionally, DDX11-AS1 has been identified as a tumor inducer for osteosarcoma and bladder cancer [14,19]. Nonetheless, the functional contributions and mechanistic basis of DDX11-AS1 have not yet been determined in glioma, which requires further exploration.

Therefore, this study aimed to characterize the oncogenic functions and molecular pathways regulated by DDX11-AS1 in glioma. In this study, we comprehensively evaluated the role of lncRNA DDX11-AS1 in glioma development and suggested a novel DDX11-AS1/miR-1183/E2F7 axis in the pathogenesis of glioma, providing us with a potential therapeutic target in glioma.

## 2 Materials and Methods

### 2.1 Animal Studies

6-week-old male immunodeficient NOD/SCID nude mice (BALB/cJ background), which were obtained from Gempharmatech Company (Nanjing, China), were used. All mice were housed under controlled conditions (23°C, 12-h light/dark cycle) with ad libitum access to sterilized water and standard diet. Investigators were aware of group assignments during experiments. Orthotopic implantation of firefly-luciferase-labeled U87 cells into the hippocampus of nude mice (*n* = 6 per experimental group) was conducted according to our established protocol [20,21]. Tumor progression was determined weekly using IVIS Spectrum *in vivo* imaging system (PerkinElmer, Waltham, MA, USA). Tumor size was quantified by total photon flux (photons/sec/cm^2^/sr), which correlates with viable tumor cell burden. Prior to imaging, mice received intraperitoneal luciferin (150 mg/kg; catalog #P1043; Promega, Madison, WI, USA) to enable photon capture. All animal protocols were approved by the Animal Experimentation Ethics Committee of Shenzhen Hospital, Southern Medical University (No. 2021-0095) and performed in accordance with institutional guidelines for animal welfare.

### 2.2 Tissue Samples

Ethical approval was obtained from the Human Ethics Committees of Shenzhen Hospital, Southern Medical University (No. NYSZYYEC20190006). All patients or their relatives provided written informed consent for this research, which was carried out in compliance with the Helsinki Declaration. All individual information was strictly kept confidential and anonymous in the manuscript. Thirty-two tissue samples of glioma and twelve samples pertaining to normal controls were collected. Clinical characteristics, prognosis and treatment history of patients with glioma were shown in Table S1. Confirmation of all glioma tissues was provided by three pathologists who worked independently, and the samples thus obtained were immediately preserved in a −80°C cryogenic refrigerator.

### 2.3 Bioinformatics Analysis

The UCSC XENA platform (https://xenabrowser.net/datapages/, accessed on 25 July 2025) is a transcripts per million databases of The Cancer Genome Atlas (TCGA, www.cancergenome.nih.gov) and Genotype-Tissue Expression (GTEx, gtexportal.org/home), processed in unison by the Toil process [22]. Based on the UCSC XENA data, lncRNA DDX11-AS1 expression between glioma and normal tissues was determined in R 3.6.3 and visualized with ‘ggplot2’ v3.3.x. Survival analysis data were downloaded from the Chinese Glioma Genome Atlas (CGGA, http://www.cgga.org.cn/) and the Gene Expression Profiling Interactive Analysis Database (http://gepia.cancer-pku.cn/) [23]. The binding site of miR-1183 in the sequence of DDX11-AS1 was analyzed by using the interactive-prediction RNA22 tool. The binding site of miR-1183 in E2F7 was predicted by bioinformatics analysis using TargetScan (http://www.targetscan.org/). Correlation analysis between DDX11-AS1 and miR-1183 expression levels in the clinical samples was subsequently performed using Spearman’s rank correlation.

### 2.4 Cell Culture and Transfection

Human glioma cell lines U87 MG (ATCC; glioblastoma of unknown origin; catalog #TCHu138; National Collection of Authenticated Cell Cultures, Shanghai, China), U251 MG (catalog #TCHu58; National Collection of Authenticated Cell Cultures), T98G (catalog #ORC0333; ORiCells Biotechnology, Shanghai, China) and U373 MG (catalog #YC-A085; UBIGENE, Guangzhou, China) were used. Human astrocyte HEB cells were used (catalog #XY-H539, X-Y Biotechnology, Shanghai, China). Lentiviral particles were produced in HEK 293T cells (catalog #YC-A007; UBIGENE). All cell cultures underwent validation via short tandem repeat profiling and confirmed absence of mycoplasma contamination. miR-1183 mimics (catalog #HY-R00073; MCE, Monmouth Junction, NJ, USA), miRNA negative controls (miR-NC; catalog #HY-R04602; MCE) and miR-1183 inhibitor (catalog #HY-RI00073; MCE) were acquired from MCE. E2F7 overexpression plasmid and corresponding empty vector control (pcDNA3.1 backbone) were synthesized by GeneChem (Shanghai, China). Cells were transiently transfected with indicated miRNA, inhibitor, or plasmid using Lipofectamine 3000 reagent (catalog #L3000015; Thermo Fisher Scientific, Waltham, MA, USA) diluted in Opti-MEM (catalog #L31985070; Thermo Fisher Scientific).

### 2.5 Generation of Stable Cell Lines with DDX11-AS1 Knockdown and Overexpression

Lentiviral vectors expressing shRNA targeting DDX11-AS1 (sh-DDX11-AS1) or negative control shRNA (sh-NC), DDX11-AS1 overexpression lentiviral vector and GV492 empty vector were designed by Genechem. The sequences for shRNA targeting DDX11-AS1 were as follows: 5^′^-GGCCGTCTAGATGACGAGTTT-3^′^. The sequences for the negative control shRNA were as follows: 5^′^-GATCGTGACTAGCTGACGTGC-3^′^. To package the lentivirus, 293T cells were transfected with the transfer vector, psPAX2 packaging plasmid, and pMD2.G envelope plasmid using Lipofectamine 3000 reagent (catalog #L3000015; Thermo Fisher Scientific) and Opti-MEM (catalog #L31985070; Thermo Fisher Scientific) as previously described [20,21]. Viral supernatant was harvested 48h post-transfection, filtered (0.45 μm), and concentrated using Ultracentrifuge (catalog #436C Optima XPN; Beckman, Brea, CA, USA). Lentiviral transduction of U87 and U251 cells was performed using 3 μg/mL polybrene (catalog #TR1003; Sigma-Aldrich, St. Louis, MO, USA) as a transduction enhancer. Puromycin (catalog #A1113803; Thermo Fisher Scientific) selection was initiated 48 h post-transduction. The efficiency of transfection was determined via Reverse Transcription Quantitative Real-time Polymerase Chain Reaction (RT-qPCR) analysis.

### 2.6 RT-qPCR

Total RNA was extracted from human tissues, U87 and U251 cells utilizing TRIzol (catalog #15596018; Thermo Fisher Scientific). Complementary DNA synthesis was achieved through the utilization of the PrimeScript RT Reagent Kit (catalog #RR037A; Takara, Kusatsu, Japan). Gene expression quantification was performed with RT-qPCR system (Applied Biosystems 7500, Foster City, CA, USA) using SYBR-Green (catalog #AQ131-02; Trans, Beijing, China) and target-specific primers. Gene expression level was quantified using the 2^−ΔΔCt^ method. Each gene was analyzed in triplicate and normalized to the control gene *U6* or *GAPDH*. Correlation analysis between DDX11-AS1 and miR-1183 expression levels in the clinical samples was subsequently performed using Spearman’s rank correlation. The primers were shown in Table S2.

### 2.7 Immunoblotting

Total protein was solubilized from the U87 and U251 cells using Radio-Immunoprecipitation Assay buffer (catalog #9803; Thermo Fisher Scientific) supplemented with a protease inhibitor cocktail (catalog #DI101-01; Trans). Extracted protein concentration was determined using a bicinchoninic acid assay (catalog #A55864; Thermo Fisher Scientific) following the manufacturer’s protocol (catalog #23227; Thermo Fisher Scientific). Protein was separated by SDS-PAGE and followed by transfer using a polyvinylidene difluoride membrane (catalog #IPVH00010; Merck, Darmstadt, Germany). Membranes was blocked using powdered milk (5%) in Tris-buffered saline comprising Tween 20 (0.1%) at room temperature for 1 h. Probing of the membranes was performed using rabbit anti-human E2F7 (1:1000; catalog #24489-1-AP; Proteintech, Rosemont, IL, USA) and rabbit anti-human GAPDH (1:5000; catalog #2118; Cell Signaling Technology, Danvers, MA, USA) at 4°C overnight. Afterwards, the membranes were subjected to incubation with anti-rabbit secondary antibody (1:5000; catalog #7074S; Cell Signaling Technology) at room temperature for 1 h. Image quantification was acquired with the 4.4.0 version of Quantity One software (Bio-Rad, Hercules, CA, USA). The experiment was repeated three times for each group.

### 2.8 Cell Counting Kit-8 (CCK-8) Assay

Evaluation of the cell proliferation was achieved using a CCK-8 assay kit (catalog #CK04-05; Dojindo, Kumamoto, Japan) according to the manufacturer’s instructions. U87 and U251 cells (5 × 10^3^ cells/well) were seeded into the 96-well plates, and 10 μL of CCK8 reagent was added into each well. Absorbance at 450 nm was then measured using the microplate reader (catalog #Varioskan ALF; Thermo Fisher Scientific).

### 2.9 Colony Formation Assay

U87 and U251 cells (1000 cells per well) were plated in 6-well plates and maintained in a medium containing 10% Fetal Bovine Serum (FBS; catalog #A5256701; Thermo Fisher Scientific) for 12 days. After washing with PBS (1.06 mM KH2PO4, 155.17 mM NaCl, 2.97 mM Na2HPO4, pH 7.4) and fixing with methanol, cells were stained with 0.5% (w/v) Crystal violet (catalog #V5265; Sigma-Aldrich). Visible colonies were determined by microscopy (DMiL LED, Leica, Wetzlar, Germany).

### 2.10 Wound Healing Assay

U87 and U251 cells (1 × 10^5^ cells per well) were plated into 6-well plates and were maintained till fully confluent. Cell monolayers were scraped in a straight line with a pipette tip. Replacement of the medium using serum-free medium was arranged. The migration distance of the cells was determined using microscopy (DMiL LED, Leica, Wetzlar, Germany) of the wounds after 24 h. The experiment was repeated three times.

### 2.11 Transwell Assay

U87 and U251 cells (1 × 10^5^) were suspended in the medium without FBS and were cultured on the top chamber of the transwell insert (catalog #CLS3464; Corning, CA, USA). The lower compartment received a chemoattractant consisting of medium containing 10% FBS. Cells were permitted to traverse the 8-μm pore polyethylene terephthalate membrane. After 24 h, cells in the lower chamber were fixed using 100% methanol and stained with 0.5% (w/v) crystal violet (catalog #V5265; Sigma-Aldrich), followed by microscopy analysis. The experiment was repeated three times.

### 2.12 Fluorescence In Situ Hybridization (FISH)

DDX11-AS1 and miR-1183 detection probes were designed and synthesized by Servicebio (Wuhan, China). Paraffin sections of human and mouse glioma were dehydrated in xylene, then covered with hybridization buffer (catalog #G3045; Servicebio). Probe hybridization proceeded at 55°C for 16 h. Sections were washed twice in 2× saline sodium citrate (SSC, catalog #G3015; Servicebio), twice in 1× SSC and once in 0.5× SSC. After washing, sections were mounted with antifluorescence tablets (catalog #G1401; Servicebio) and then visualized by microscopy (DM4B, Leica).

### 2.13 Luciferase Activity Assay

U87 glioma cells were used. The 3^′^UTR sequences of the wild-type (WT) DDX11-AS1, mutant (MUT) DDX11-AS1, WT E2F7, or MUT E2F7 were constructed in the pGL3 luciferase vector (Genechem, Shanghai, China). Lipofectamine 3000 (catalog #L3000015; Thermo Fisher Scientific) was used for the transfection of the indicated luciferase vectors and miRNAs. The luciferase activity was determined by the luciferase reporter assay kit (catalog #FR201; Trans) as we previously described [20,21].

### 2.14 RNA Immunoprecipitation (RIP) Assay

The RIP assay was performed using a Magna RNA immunoprecipitation kit (catalog #17-700; Sigma-Aldrich). In brief, U87 and U251 cells were lysed using RIP lysis buffer. Then, magnetic beads were used and incubated with anti-Ago2 antibody (1:50, catalog #67934-1-Ig; MilliporeSigma, Burlington, MA, USA) or a negative control IgG antibody (1:50, catalog #ab172730; Abcam, Cambridge, UK). Magnetic beads bound complexes were were isolated magnetically, with non-specifically bound materials eliminated by sequential washes. The coprecipitated RNAs were then extracted and measured by RT-qPCR.

### 2.15 miRNA-Pulldown Assay

U87 and U251 cells were transfected with biotin-labeled miR-NC and miR-1183 (100 nM). The cells were collected and lysed in cold lysis buffer supplemented with RNase inhibitor (catalog #R0102; Beyotime, Shanghai, China) at 48 h post-transfection. Streptavidin-Dyna beads (catalog #11205D, Thermo Fisher Scientific) were pre-coated with yeast tRNA (catalog #AM7119, Thermo Fisher Scientific) and Bovine Serum Albumin (catalog #ST025; Beyotime) in the lysis buffer with rotation at 4°C for 1 h. The lysates were incubated with pre-coated beads at 4°C for 4 h with rotation. The beads were washed six times with cold lysis buffer. Bound RNA was extracted by adding TRIzol reagent directly to beads, followed by chloroform purification and ethanol precipitation. DDX11-AS1 expression in pulldown complexes was quantified via RT-qPCR, with data calibration against the endogenous control *GAPDH*.

### 2.16 Statistics Analysis

Sample size calculations incorporated statistical power analysis derived from prior studies and preliminary data variability. Results are expressed as the mean ± standard error of the mean (SEM) using Prism 10.0 (GraphPad Software, San Diego, CA, USA). An unpaired student’s *t*-test was done to compare the two groups. Comparisons among multiple groups were done by using one-way ANOVA with Bonferroni correction for multiple comparisons. The statistical significance for the survival curve was calculated using the long-rank test. Statistical significance was indicated for any difference where the *p*-value was less than 0.05.

## 3 Results

### 3.1 Elevated lncRNA DDX11-AS1 Expression in Human Glioma Specimens

**Figure 1:**
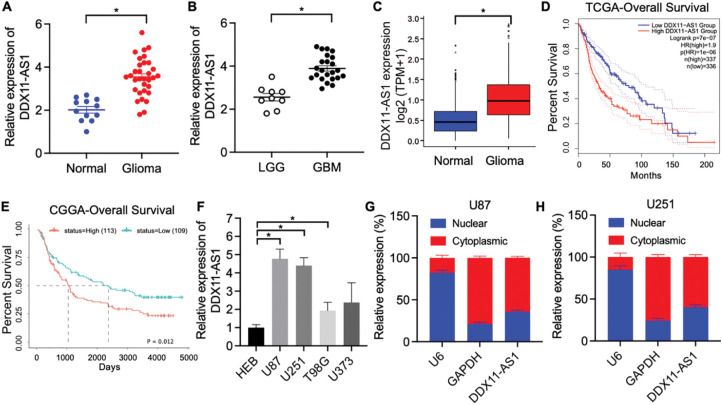
lncRNA DDX11-AS1 expression is upregulated in human glioma specimens. (**A**) Expression of lncRNA DDX11-AS1 was evaluated in 32 glioma tissues and 12 normal brain tissues by RT-qPCR. (**B**) Expression of lncRNA DDX11-AS1 was identified in 9 low-grade glioma (LGG) tissues and 23 glioblastoma (GBM) tissues by RT-qPCR. (**C**) lncRNA DDX11-AS1 expression level in open-access datasets of TCGA and GTEx (*n* = 1152 for normal brain tissues and *n* = 689 for glioblastoma). (**D**) Kaplan-Meier plots of overall survival in glioma patients with high (*n* = 337) and low (*n* = 336) levels of DDX11-AS1 in the TCGA database. (**E**) Kaplan-Meier plots of overall survival in glioma patients with high (*n* = 113) and low (*n* = 109) levels of DDX11-AS1 in the CGGA database. (**F**) Expression of lncRNA DDX11-AS1 in HEB cells and four glioma cell lines (*n* = 6). (**G**,**H**) Levels of DDX11-AS1 in the nuclear and cytoplasmic fractions of U87 and U251 cells (*n* = 6). Data are expressed as the mean ± SEM, with statistical significance (*p < 0.05) determined by Student’s *t* test

To elucidate the pathophysiological contribution of lncRNA DDX11-AS1 in human glioma, we quantitatively measured its expression in clinical human glioma specimens and normal brain tissues obtained from surgical patients. RT-qPCR results showed that the expression of DDX11-AS1 was significantly higher in glioma tissues, especially increased with advanced glioma grades (Fig. 1A,B). Consistently, the expression of lncRNA DDX11-AS1 was up-regulated in glioma as revealed by gene expression analysis of TCGA and GTEx databases (Fig. 1C). TCGA-based Kaplan-Meier assessment associated elevated DDX11-AS1 expression with markedly reduced patient overall survival (Fig. 1D). Validation in an independent cohort from the CGGA database further corroborated these observations (Fig. 1E). Furthermore, HEB cells and the four types of glioma cells were subjected to RT-qPCR analysis for DDX11-AS1 expression. Among the four glioma cell lines, U87 and U251 exerted the highest expression and were chosen for all subsequent experiments (Fig. 1F). Next, RT-qPCR analysis of nuclear and cytoplasmic RNAs was conducted to identify the cellular localization of DDX11-AS1. The result indicated predominant cytoplasmic localization of DDX11-AS1 in U87 and U251 cells (Fig. 1G,H). Taken together, these findings indicated that DDX11-AS1 expression is significantly up-regulated in glioma specimens and might contribute to glioma development.

### 3.2 lncRNA DDX11-AS1 Promotes Glioma Proliferation and Migration In Vitro

**Figure 2:**
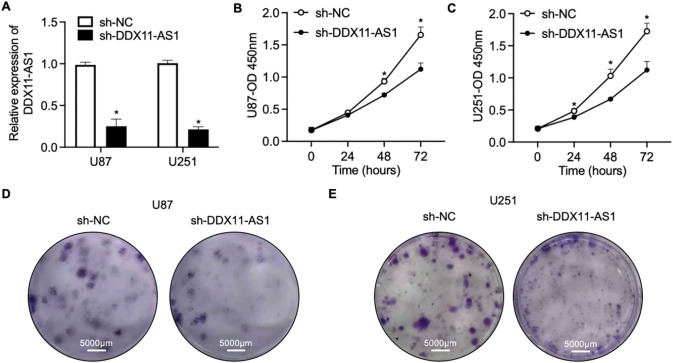

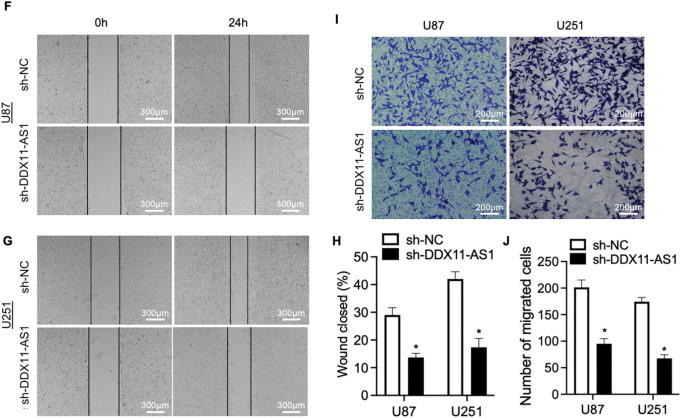
Knockdown of DDX11-AS1 suppresses the proliferation and migration in glioma cells. Glioma cells transfected with shDDX11-AS1 or sh-NC were used. (**A**) Expression level of lncRNA DDX11-AS1 in glioma cells. The expression level of sh-DDX11-AS1 U87 and U251 is normalized to *GAPDH*, and expressed as fold change over the sh-NC U87 or sh-NC U251 group, respectively (*n* = 6). (**B**,**C**) CCK-8 assay of (**B**) U87 and (**C**) U251 cells (*n* = 6). (**D**,**E**) Colony formation assay of (**D**) U87 and (**E**) U251 cells (*n* = 6). (**F**,**G**) Representative images of scratch wound healing assay of (**F**) U87 and (**G**) U251 cells. (**H**) The percentage of wound closure at 24 h (*n* = 6). (**I**) Transwell assay of U87 and U251 cells. (**J**) Quantified data depicting migrated cell number (*n* = 6). Data are expressed as the mean ± SEM, with statistical significance (*p < 0.05) determined by Student’s *t* test

**Figure 3:**
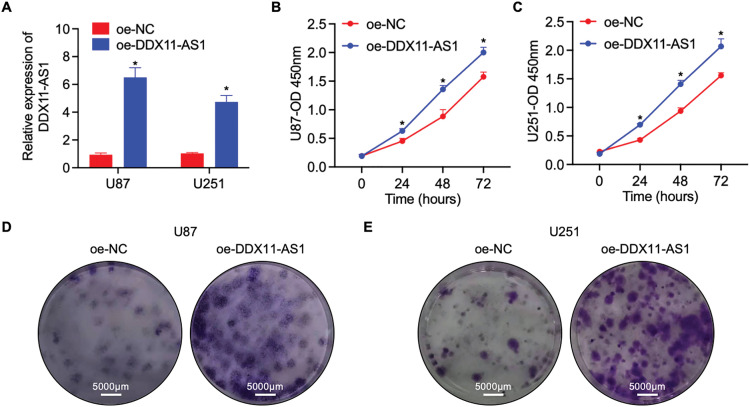

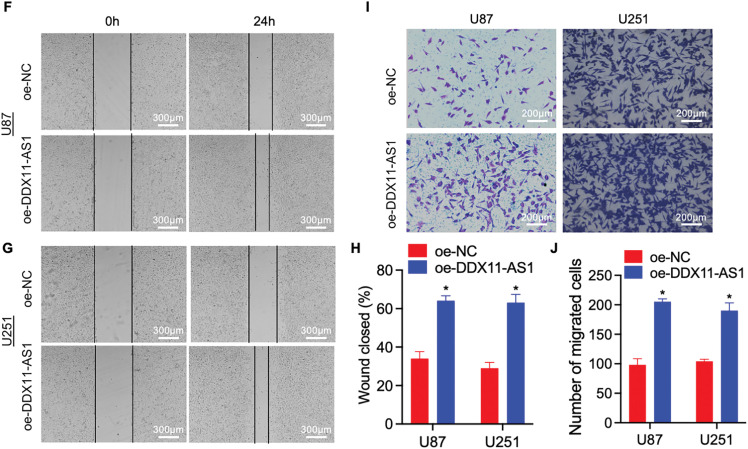
Overexpression of DDX11-AS1 promotes the proliferation and migration in glioma cells. Glioma cells with DDX11-AS1 overexpression or harboring the empty vector were used. (**A**) Expression level of lncRNA DDX11-AS1 in glioma cells. The expression level of oe-DDX11-AS1 U87 and U251 is normalized to *GAPDH*, and expressed as fold change over the oe-NC U87 or oe-NC U251 group, respectively (*n* = 6). (**B**,**C**) CCK-8 assay of (**B**) U87 and (**C**) U251 cells (*n* = 6). (**D**,**E**) Colony formation assay of (**D**) U87 and (**E**) U251 cells (*n* = 6). (**F**,**G**) Representative scratch assay images of (**F**) U87 and (**G**) U251 cells. (**H**) The percentage of wound closure at 24 h (*n* = 6). (**I**) Transwell migration analysis of U87 and U251 cells. (**J**) Quantified data depicting migrated cell number (*n* = 6). Data are expressed as the mean ± SEM, with statistical significance (*p < 0.05) determined by Student’s *t* test

To elucidate the modulatory function of DDX11-AS1 in gliomagenesis, we implemented a lentiviral-mediated shRNA silencing approach to knock down the DDX11-AS1 expression in U87 and U251 glioma cells. The knockdown efficiency of the DDX11-AS1 was quantified as ~27% in U87 cells and ~22% in U251 cells, as measured by RT-qPCR (Fig. 2A). CCK-8 and colony formation assays revealed that knockdown of DDX11-AS1 suppressed the proliferation in glioma cells (Fig. 2B–E). Migration capacity of glioma cells was further determined by wound healing and transwell assay. These findings demonstrated that subsequent to DDX11-AS1 knockdown, glioma cell migration capacity was significantly attenuated relative to control groups (Fig. 2F–J). To further delineate the functional impact of DDX11-AS1 in glioma cells, DDX11-AS1 was overexpressed by transfection of pcDNA3.1-DDX11-AS1 plasmids into the U87 and U251 glioma cells. DDX11-AS1 overexpression was confirmed with the RT-qPCR (Fig. 3A). In contrast to knockdown, overexpression of DDX11-AS1 enhanced proliferation and migration of glioma cells (Fig. 3B–J). These results illuminated that DDX11-AS1 promotes glioma proliferation and migration capacity *in vitro*.

### 3.3 lncRNA DDX11-AS1 Drives Gliomagenesis In Vivo

**Figure 4:**
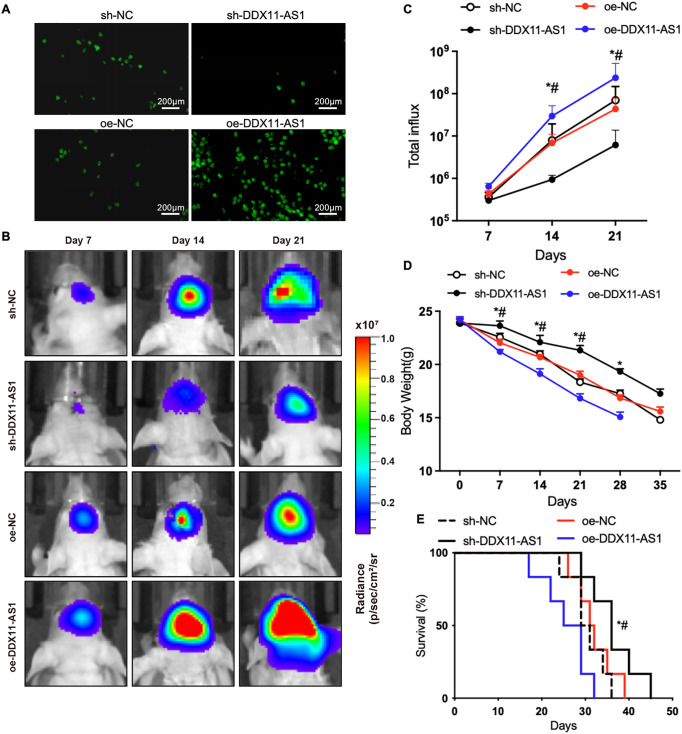
lncRNA DDX11-AS1 promotes orthotopic glioma growth and shortens lifespan in mice. (**A**) RNA FISH for DDX11-AS1 in orthotopic glioma samples after inoculation with glioma cells. (**B**) Longitudinal bioluminescence monitoring of tumor-bearing nude mice. (**C**) The quantification of luminescence signal intensity (*n* = 6). (**D**) Body weight of mice (*n* = 6). All data are represented as the mean ± SEM, *p < 0.05, sh-DDX11-AS1 vs. sh-NC; ^#^*p* < 0.05, oe-DDX11-AS1 vs. oe-NC (One-way ANOVA). (**E**) Survival curve of mice. *p < 0.05, sh-DDX11-AS1 vs. sh-NC; ^#^*p* < 0.05, oe-DDX11-AS1 vs. oe-NC (Long-rank test)

Next, to investigate DDX11-AS1’s oncogenic functions *in vivo*, orthotopic glioma-bearing mice were used. Orthotopic glioma-bearing mice were generated by orthotopically implanting firefly-luciferase-labeled U87 cells into the hippocampal parenchyma of immunocompromised mice. RNA FISH for DDX11-AS1 in orthotopic glioma samples after inoculation with U87 cells indicated that the knockdown and overexpression of DDX11-AS1 *in vivo* had the same results as *in vitro* cell culture (Fig. 4A). Glioma cells transduced with lentiviral sh-DDX11-AS1 inhibited gliomagenesis in nude mice as quantified through *in vivo* bioluminescent imaging (Fig. 4B,C). Moreover, sh-DDX11-AS1 glioma cells slowed down the body weight loss and extended median survival of mice (Fig. 4D,E). In contrast to glioma cells with DDX11-AS1 knockdown, glioma cells with DDX11-AS1 overexpression promoted tumor growth and reduced overall survival of nude mice (Fig. 4B–E). Collectively, these results strongly suggested that DDX11-AS1 is a potential tumor inducer in glioma malignant development.

### 3.4 DDX11-AS1 Functions as a Competing Endogenous RNA (ceRNA) for miR-1183 in Glioma

**Figure 5:**
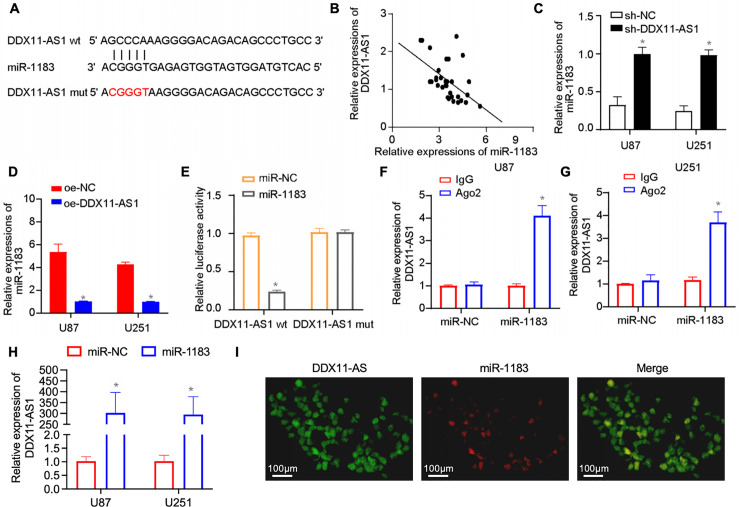
DDX11-AS1 competitively binds miR-1183 through competing endogenous RNA (ceRNA) mechanism. (**A**) *In silico* analysis of the binding site of miR-1183 within DDX11-AS1 sequence. (**B**) Correlation between DDX11-AS1 and miR-1183 expression in clinical glioma samples (*n* = 32). (**C**,**D**) The expression level of miR-1183 in glioma cells with DDX11-AS1 (**C**) knockdown or (**D**) overexpression (*n* = 6). (**E**) The relative luciferase activity was examined in U87 glioma cells co-transfected with DDX11-AS1 wild-type (WT) or DDX11-AS1 mutant (MUT) and miR-1183 mimics or the miR-NC. (**F**,**G**) RIP assay in (**F**) U87 and (**G**) U251 glioma cells. (**H**) Binding of miR-1183 and miR-NC to DDX11-AS1 in the RNA pull-down assay (GAPDH as control). (**I**) RNA FISH for DDX11-AS1 and miR-1183 in human glioma sample. Data are expressed as the mean ± SEM, with statistical significance (*p < 0.05) determined by Student’s *t* test

Cytoplasmic lncRNAs can sequester miRNAs to modulate downstream pathways [24–26]. *In silico* prediction identified DDX11-AS1 as a ceRNA for miR-1183, supported by their inverse correlation in glioma specimens (Fig. 5A,B). Knockdown of DDX11-AS1 increased the expression of miR-1183, whereas its overexpression resulted in significant down-regulation of miR-1183 (Fig. 5C,D). Then, the construction of mutated or wild-type miR-1183 binding sites in DDX11-AS1 was achieved (Fig. 5A), followed by luciferase reporter vector insertion to validate the direct binding between miR-1183 and DDX11-AS1. Luciferase reporter assay showed that miR-1183 dramatically suppressed the luciferase activity of DDX11-AS1 WT but had no effect on the DDX11-AS1 MUT, suggesting that miR-1183 was a target of DDX11-AS1 in a sequence-specific manner (Fig. 5E). Ago2 immunoprecipitation assay revealed that miR-1183 and DDX11-AS1 were both enriched in the Ago2 IP (Fig. 5F,G). In addition, it was found that biotin-labeled miR-1183 pulled down DDX11-AS1 in U87 and U251 cells, indicating that miR-1183 may directly bind to DDX11-AS1 (Fig. 5H). RNA FISH for DDX11-AS1 and miR-1183 in human glioma samples further indicated the interaction between DDX11-AS1 and miR-1183 (Fig. 5I). Overall, these results outlined above demonstrated that DDX11-AS1 functions as a ceRNA for miR-1183 in glioma.

### 3.5 DDX11-AS1 Promotes the Proliferation and Migration of Glioma Cells by Targeting the miR-1183/E2F7 Axis

**Figure 6:**
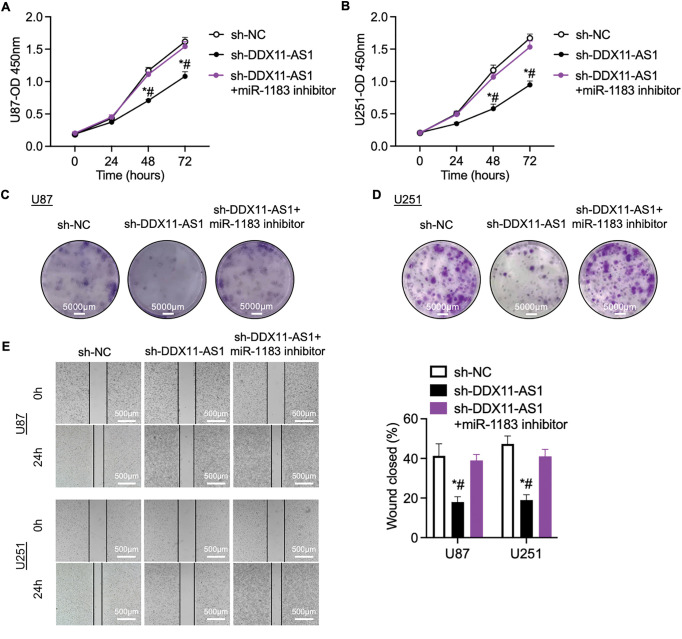

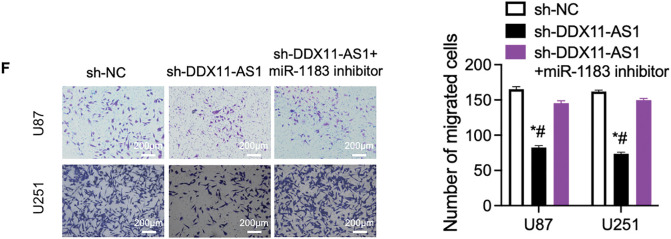
DDX11-AS1 promotes glioma cell proliferation and migration by targeting miR-1183. Glioma cells transfected with sh-NC, sh-DDX11-AS1 or sh-DDX11-AS1 followed by miR-1183 inhibitor treatment were used. (**A**,**B**) CCK-8 assays of (**A**) U87 and (**B**) U251 glioma cells (*n* = 6). (**C**,**D**) Colony formation assays of (**C**) U87 and (**D**) U251 glioma cells (*n* = 6). (**E**) Scratch wound healing assay of U87 and U251 glioma cells. (**F**) Transwell assay of U87 and U251 glioma cells. The right panel is the quantified data depicting migrated cell number (*n* = 6). All data are represented as the mean ± SEM, *p < 0.05, sh-DDX11-AS1 vs. sh-NC; ^#^*p* < 0.05, sh-DDX11-AS1 vs. sh-DDX11-AS1 + miR-1183 inhibitor (One-way ANOVA)

To validate the role of miR-1183 in glioma progression, glioma cells with DDX11-AS1 silencing were treated with miR-1183 inhibitors. The suppression of cell proliferation induced by sh-DDX11-AS1 in glioma cells was recovered by miR-1183 inhibitor, as revealed by CCK-8 and colony formation assays (Fig. 6A–D). In addition, wound healing and transwell assays showed that the miR-1183 inhibitor rescued the migratory activity of sh-DDX11-AS1 glioma cells (Fig. 6E,F). Collectively, these results demonstrated that DDX11-AS1 promoted the proliferation and migration of glioma cells in a miR-1183-dependent manner.

**Figure 7:**
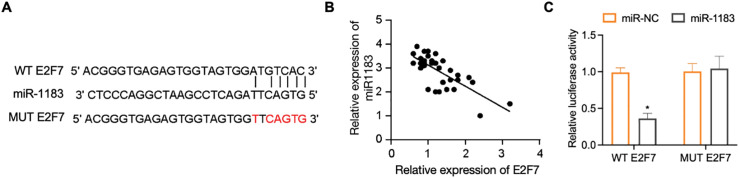

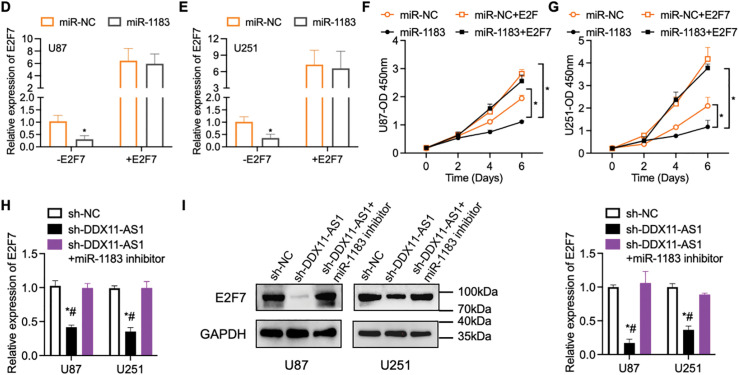
E2F7 is a target of miR-1183. (**A**) The bioinformatics analysis tools were used to predict the binding site of miR-1183 in E2F7. (**B**) Correlation between miR-1183 and E2F7 expression. (**C**) Luciferase activity in U87 cells co-transfected with miR-1183 and WT/MUT E2F7 reporter constructs (*n* = 6). (**D**–**G**) U87 and U251 cells transfected with miR-1183, miR-NC or E2F7-overexpression vectors were used; (**D**,**E**) Relative expression of *E2F7* was determined by RT-qPCR (*n* = 6); (**F**,**G**) CCK-8 assay of (**F**) U87 and (**G**) U251 cells (*n* = 6); (**H**,**I**) U87 and U251 cells transfected with sh-NC, sh-DDX11-AS1 or sh-DDX11-AS1 followed with miR-1183 inhibitor treatment were used. (**H**) Relative expression of *E2F7* was determined by RT-qPCR (*n* = 6). (**I**) Immunoblotting assays detected E2F7 and GAPDH in U87 and U251 cells. The bar chart quantifies relative E2F7 protein abundance normalized to GAPDH (*n* = 6). All data are represented as the mean ± SEM, *p < 0.05, sh-DDX11-AS1 vs. sh-NC; ^#^*p* < 0.05, sh-DDX11-AS1 vs. sh-DDX11-AS1 + miR-1183 inhibitor (One-way ANOVA)

To further delineate the regulatory mechanism of DDX11-AS1/miR-1183 in glioma development, we performed a bioinformatics analysis and found that miR-1183 could potentially bind to the *E2F7* mRNA 3^′^UTR region (Fig. 7A). E2F7 was reported to be a well-established glioma oncogene [27,28]. Clinically, E2F7 transcript levels exhibited inverse correlation with miR-1183 abundance in glioma specimens (Fig. 7B). To verify whether miR-1183 regulates E2F7 expression by binding to its 3^′^UTR, we mutated the 3^′^UTR of *E2F7* and performed a firefly/renilla dual-luciferase reporter assay. miR-1183 mimics suppressed E2F7 WT 3^′^UTR reporter activity but had no effect on E2F7 MUT, as confirmed by dual-luciferase assays (Fig. 7C). Furthermore, E2F7 overexpression largely rescued the miR-1183-mediated suppression of glioma cell proliferation (Fig. 7D–G). Consistent with this, knockdown of DDX11-AS1 down-regulated both mRNA and protein levels of E2F7, while such effect was rescued by miR-1183 inhibitor (Fig. 7H,I), suggesting that miR-1183 regulates *E2F7* expression by binding to its 3^′^UTR. In conclusion, our findings illustrated that DDX11-AS1 promoted glioma progression, which was primarily dependent on the miR-1183/E2F7 axis.

## 4 Discussion

Growing evidence suggests the essential roles of lncRNAs in pathological processes across malignancies, with glioma representing a critical context [29,30]. For instance, lncRNA NEAT1 enhances the growth and invasiveness of glioma cells by modulating the miR-132/SOX2 axis [31]. Moreover, lncRNA TALC functions as an important mediator, regulating the c-Met signaling pathway and promoting the expression of MGMT. Targeting lncRNA TALC in glioma can overcome temozolomide resistance and recurrence [25]. Recent investigations increasingly point to a role for DDX11-AS1 in governing the progression of various malignancies [13–15]. However, the specific functions and mechanistic underpinnings of DDX11-AS1 in glioma are still unclear. In this study, we demonstrated that the expression of DDX11-AS1 was significantly increased in glioma tissues and was positively correlated with glioma grade. These results indicated that DDX11-AS1 might play as an oncogenic driver during glioma progression. The loss- and gain-of-function assays revealed that DDX11-AS1 remarkably promotes malignant phenotypes of glioma *in vitro* and *in vivo*. Mechanistically, we demonstrated that DDX11-AS1 acted as a molecular sponge to modulate the expression of E2F7 through sponging miR-1183. This work provides the first comprehensive elucidation of the functional roles and molecular mechanisms of DDX11-AS1 in glioma. These findings suggested that DDX11-AS1 functions as an oncogene that promotes glioma development and can be a therapeutic target for glioma treatment.

Growing evidence has demonstrated that lncRNAs exert oncogenic or tumor-suppressive functions in cancer development by sponging miRNAs [24–26]. miR-1183 was shown to be dysregulated in several human diseases, including mesial temporal lobe epilepsy, intracerebral hemorrhage and rheumatic heart disease [32–34]. In addition, miR-1183 drives tumorigenesis of non-small cell lung cancer [35]. However, its regulatory role in glioma has not been studied. Crucially, our study advances the field by identifying miR-1183 as the novel, central miRNA component of the DDX11-AS1 ceRNA axis specific to glioma pathogenesis. Prior studies have linked DDX11-AS1 to other miRNAs in different cancer types, but the discovery of its direct interaction with, and sponging of, miR-1183 within glioma cells and tissues constitutes a key mechanistic novelty of this work. This specific DDX11-AS1/miR-1183/E2F7 axis defines a previously unrecognized regulatory pathway underlying glioma progression.

In this study, we identified that miR-1183 binds to the 3^′^UTR of *E2F7* in a direct fashion. E2F7 was shown to function as a tumor inducer in several cancer types, including lung cancer, hepatocellular cancer and gallbladder cancer [36–38]. The tumor-promoting effect of E2F7 was also reported in glioma. Specifically, E2F7 promotes tumorigenesis via EZH2-mediated PTEN/AKT/mTOR pathway in glioma [28]. Although the PTEN/AKT/mTOR axis downstream of E2F7 is well-established, its future exploration in our specific context remains critical to elucidate underlying mechanisms. Here, miR-1183 was shown to directly target E2F7, exerting a tumor-promoting role in glioma. Furthermore, DDX11-AS1 knockdown suppressed E2F7 expression, an effect reversed by miR-1183 inhibition, indicating that DDX11-AS1 positively regulates E2F7 through miR-1183 sponging. Emerging evidence, however, suggests DDX11-AS’s functions extend beyond miRNA sponging. Systematic mapping of the interactome of DDX11-AS1 using RIP-seq or ChIRP-seq could identify novel RNA-binding proteins and chromatin regulators. In addition, future studies should further investigate whether DDX11-AS1 orchestrates spatial genome architecture through cohesin-mediated chromatin loop formation, a mechanism implicated in DDX11-associated cohesinopathies. Such exploration may uncover context-dependent oncogenic networks and guide the development of combinatorial therapies targeting lncRNA-protein complexes.

Beyond cell-autonomous oncogenic mechanisms that drive tumor proliferation and invasion, the immune microenvironment critically contributes to cancer progression [39,40]. In our study, we elucidated the cell-autonomous oncogenic role of the DDX11-AS1/miR-1183/E2F7 axis in promoting glioma proliferation and invasion. However, a key limitation arises from the absence of functional immune components in our immunodeficient orthotopic model, which precludes a comprehensive investigation of immune-tumor crosstalk. To address this, syngeneic models (e.g., GL261/CT2A in C57BL/6 mice) or humanized mouse systems could enable deeper exploration into the dynamics of the glioma immune microenvironment. Another limitation is the lack of assessment of whether conventional therapeutics confound DDX11-AS1 expression patterns. Since the extremely limited number of pretreated recurrent cases (*n* = 1 chemotherapy-only; *n* = 2 combined chemo-radiotherapy), we are unable to perform a robust analysis to determine whether chemotherapy and/or radiotherapy exposure influenced DDX11-AS1 expression levels. Future studies with expanded cohorts receiving standardized neoadjuvant/adjuvant regimens are needed to dissect potential treatment-induced modulation of DDX11-AS1. Furthermore, the therapeutic implications of DDX11-AS1-targeting antisense oligonucleotides (ASOs) warrant prioritization in future investigations, as no *in vivo* efficacy testing has been conducted. While direct *in vivo* validation would substantially strengthen our mechanistic findings, such studies face significant technical constraints, primarily due to the limited blood-brain barrier (BBB) penetrability of systemically administered ASOs [41]. Nevertheless, emerging delivery oligonucleotide transport vehicles offer potential to enhance ASO bioavailability for future glioma therapeutics [42,43].

In conclusion, this study indicated that lncRNA DDX11-AS1 plays an important role in the pathogenesis of human glioma. DDX11-AS1 expression is significantly elevated in glioma tissues and is positively correlated with glioma grade. DDX11-AS1 promotes glioma development *in vitro* and *in vivo*. Mechanistically, DDX11-AS1 competitively binds with miR-1183, in turn, promoting the proliferative and migratory actions of glioma cells through inducing E2F7 expression. Therefore, our study provides a valuable and potential biomarker and a therapeutic target for glioma.

## Supplementary Materials



## Data Availability

Data supporting the findings of this study are available from the corresponding authors upon request.

## Supplementary Materials

The supplementary material is available online at https://www.techscience.com/doi/10.32604/or.2025.065380/s1.

## References

[1] Schaff LR, Mellinghoff IK. Glioblastoma and other primary brain malignancies in adults: a review. JAMA. 2023;329(7):574–87. doi:10.1001/jama.2023.0023; 36809318 PMC11445779

[2] Girardi F, Matz M, Stiller C, You H, Marcos Gragera R, Valkov MY, et al. Global survival trends for brain tumors, by histology: analysis of individual records for 556,237 adults diagnosed in 59 countries during 2000–2014 (CONCORD-3). Neuro-Oncol. 2023;25(3):580–92. doi:10.1093/neuonc/noac217; 36355361 PMC10013649

[3] Ben Mrid R, El Guendouzi S, Mineo M, El Fatimy R. The emerging roles of aberrant alternative splicing in glioma. Cell Death Discov. 2025;11(1):50. doi:10.1038/s41420-025-02323-0; 39915450 PMC11802826

[4] Müller Bark J, Kulasinghe A, Chua B, Day BW, Punyadeera C. Circulating biomarkers in patients with glioblastoma. Br J Cancer. 2020;122(3):295–305. doi:10.1038/s41416-019-0603-6; 31666668 PMC7000822

[5] Mattick JS, Amaral PP, Carninci P, Carpenter S, Chang HY, Chen LL, et al. Long non-coding RNAs: definitions, functions, challenges and recommendations. Nat Rev Mol Cell Biol. 2023;24(6):430–47. doi:10.1038/s41580-022-00566-8; 36596869 PMC10213152

[6] Wu J, Yu H, Huang H, Shu P, Peng X. Functions of noncoding RNAs in glial development. Dev Neurobiol. 2021;81(7):877–91. doi:10.1002/dneu.22848; 34402590

[7] Adhikary S, Singh V, Choudhari R, Yang B, Adhikari S, Ramos EI, et al. ZMYND8 suppresses MAPT213 lncRNA transcription to promote neuronal differentiation. Cell Death Dis. 2022;13(9):766. doi:10.1038/s41419-022-05212-x; 36064715 PMC9445031

[8] Li Y, Jiang T, Zhou W, Li J, Li X, Wang Q, et al. Pan-cancer characterization of immune-related lncRNAs identifies potential oncogenic biomarkers. Nat Commun. 2020;11(1):1000. doi:10.1038/s41467-020-14802-2; 32081859 PMC7035327

[9] Xu H, Zhang B, Yang Y, Li Z, Zhao P, Wu W, et al. lncRNA MIR4435-2HG potentiates the proliferation and invasion of glioblastoma cells via modulating miR-1224-5p/TGFBR2 axis. J Cell Mol Med. 2020;24(11):6362–72. doi:10.1111/jcmm.15280; 32319715 PMC7294147

[10] Dong P, Xiong Y, Yue J, Xu D, Ihira K, Konno Y, et al. Long noncoding RNA NEAT1 drives aggressive endometrial cancer progression via miR-361-regulated networks involving STAT3 and tumor microenvironment-related genes. J Exp Clin Cancer Res. 2019;38(1):295. doi:10.1186/s13046-019-1306-9; 31287002 PMC6615218

[11] Li Y, Zhou M, Yang L, Liu S, Yang L, Xu B, et al. lncRNA DDX11-AS1 promotes breast cancer progression by targeting the miR-30c-5p/MTDH axis. Sci Rep. 2024;14(1):26745. doi:10.21203/rs.3.rs-3822928/v1. 39501057 PMC11538490

[12] Xu M, Zhao X, Zhao S, Yang Z, Yuan W, Han H, et al. Landscape analysis of lncRNAs shows that DDX11-AS1 promotes cell-cycle progression in liver cancer through the PARP1/p53 axis. Cancer Lett. 2021;520(8):282–94. doi:10.1016/j.canlet.2021.08.001; 34371129

[13] Li Y, Zhuang W, Huang M, Li X. Long noncoding RNA DDX11-AS1 epigenetically represses LATS2 by interacting with EZH2 and DNMT1 in hepatocellular carcinoma. Biochem Biophys Res Commun. 2019;514(4):1051–7. doi:10.1016/j.bbrc.2019.05.042; 31097223

[14] Chen D, Chen J, Gao J, Zhang Y, Ma Y, Wei W, et al. lncRNA DDX11-AS1 promotes bladder cancer occurrence via protecting LAMB3 from downregulation by sponging miR-2355-5p. Cancer Biother Radiopharm. 2020;35(5):319–28. doi:10.1089/cbr.2019.3021; 32412777

[15] Song W, Qian Y, Zhang MH, Wang H, Wen X, Yang XZ, et al. The long non-coding RNA DDX11-AS1 facilitates cell progression and oxaliplatin resistance via regulating miR-326/IRS1 axis in gastric cancer. Eur Rev Med Pharmacol Sci. 2020;24(6):3049–61. doi:10.1080/21691401.2020.1726937; 32271422

[16] Shi M, Zhang XY, Yu H, Xiang SH, Xu L, Wei J, et al. DDX11-AS1 as potential therapy targets for human hepatocellular carcinoma. Oncotarget. 2017;8(27):44195–202. doi:10.18632/oncotarget.17409; 28496001 PMC5546473

[17] Liu H, Zhang Z, Wu N, Guo H, Zhang H, Fan D, et al. Integrative analysis of dysregulated lncRNA-associated ceRNA network reveals functional lncRNAs in gastric cancer. Genes. 2018;9(6):303. doi:10.3390/genes9060303; 29912172 PMC6027299

[18] Ren Z, Liu X, Si Y, Yang D. Long non-coding RNA DDX11-AS1 facilitates gastric cancer progression by regulating miR-873-5p/SPC18 axis. Artif Cells Nanomed Biotechnol. 2020;48(1):572–83. doi:10.1080/21691401.2020.1726937; 32054332

[19] Zhang H, Lin J, Chen J, Gu W, Mao Y, Wang H, et al. DDX11-AS1 contributes to osteosarcoma progression via stabilizing DDX11. Life Sci. 2020;254:117392. doi:10.1016/j.lfs.2020.117392; 32014424

[20] Chen T, Liu J, Wang C, Wang Z, Zhou J, Lin J, et al. ALOX5 contributes to glioma progression by promoting 5-HETE-mediated immunosuppressive M2 polarization and PD-L1 expression of glioma-associated microglia/macrophages. J Immunother Cancer. 2024;12(8):e009492. doi:10.1136/jitc-2024-009492; 39142719 PMC11332009

[21] Yang X, Liu J, Wang C, Cheng KK, Xu H, Li Q, et al. MiR-18a promotes glioblastoma development by down-regulating ALOXE3-mediated ferroptotic and anti-migration activities. Oncogenesis. 2021;10(2):15. doi:10.1038/s41389-021-00304-3; 33579899 PMC7881152

[22] Vivian J, Rao AA, Nothaft FA, Ketchum C, Armstrong J, Novak A, et al. Toil enables reproducible, open source, big biomedical data analyses. Nat Biotechnol. 2017;35(4):314–6. doi:10.1038/nbt.3772; 28398314 PMC5546205

[23] Tang Z, Li C, Kang B, Gao G, Li C, Zhang Z. GEPIA: a web server for cancer and normal gene expression profiling and interactive analyses. Nucleic Acids Res. 2017;45(W1):W98–102. doi:10.1093/nar/gkx247; 28407145 PMC5570223

[24] Mu M, Niu W, Zhang X, Hu S, Niu C. lncRNA BCYRN1 inhibits glioma tumorigenesis by competitively binding with miR-619-5p to regulate CUEDC2 expression and the PTEN/AKT/p21 pathway. Oncogene. 2020;39(45):6879–92. doi:10.1038/s41388-021-01990-4; 32978519 PMC7644463

[25] Wu P, Cai J, Chen Q, Han B, Meng X, Li Y, et al. Lnc-TALC promotes O^6^-methylguanine-DNA methyltransferase expression via regulating the c-Met pathway by competitively binding with miR-20b-3p. Nat Commun. 2019;10(1):2025. doi:10.1093/neuonc/noaa222.847. 31053733 PMC6499807

[26] Xu J, Xu J, Liu X, Jiang J. The role of lncRNA-mediated ceRNA regulatory networks in pancreatic cancer. Cell Death Discov. 2022;8(1):287. doi:10.1038/s41420-022-01061-x; 35697671 PMC9192730

[27] Meng J, Qian W, Yang Z, Gong L, Xu D, Huang H, et al. p53/E2F7 axis promotes temozolomide chemoresistance in glioblastoma multiforme. BMC Cancer. 2024;24(1):317; 38454344 10.1186/s12885-024-12017-y PMC10921682

[28] Yang R, Wang M, Zhang G, Bao Y, Wu Y, Li X, et al. E2F7-EZH2 axis regulates PTEN/AKT/mTOR signalling and glioblastoma progression. Br J Cancer. 2020;123(9):1445–55. doi:10.1038/s41416-020-01032-y; 32814835 PMC7591888

[29] Gareev I, Encarnacion Ramirez MJ, Nurmukhametov R, Ivliev D, Shumadalova A, Ilyasova T, et al. The role and clinical relevance of long non-coding RNAs in glioma. Noncoding RNA Res. 2023;8(4):562–70. doi:10.1016/j.ncrna.2023.08.005; 37602320 PMC10432901

[30] Pokorná M, Černá M, Boussios S, Ovsepian SV, O’Leary VB. lncRNA biomarkers of glioblastoma multiforme. Biomedicines. 2024;12(5):932. doi:10.3390/biomedicines12050932; 38790894 PMC11117901

[31] Zhou K, Zhang C, Yao H, Zhang X, Zhou Y, Che Y, et al. Knockdown of long non-coding RNA NEAT1 inhibits glioma cell migration and invasion via modulation of SOX2 targeted by miR-132. Mol Cancer. 2018;17(1):105. doi:10.1186/s12943-018-0849-2; 30053878 PMC6064054

[32] Antônio LGL, Freitas-Lima P, Pereira-da-Silva G, Assirati JA, Matias CM, Cirino MLA, et al. Expression of microRNAs miR-145, miR-181c, miR-199a and miR-1183 in the blood and hippocampus of patients with mesial temporal lobe epilepsy. J Mol Neurosci. 2019;69(4):580–7. doi:10.1007/s12031-019-01386-w; 31368064

[33] Li N, Zhu L, Zhou H, Zheng D, Xu G, Sun L, et al. miRNA-1183-tregulation of Bcl-2 contributes to the pathogenesis of rheumatic heart disease. Biosci Rep*,* 40(11):BSR20201573. doi:10.1042/bsr20201573; 33073840 PMC7607189

[34] Cheng X, Ander BP, Jickling GC, Zhan X, Hull H, Sharp FR, et al. MicroRNA and their target mRNAs change expression in whole blood of patients after intracerebral hemorrhage. J Cereb Blood Flow Metab. 2020;40(4):775–86. doi:10.1177/0271678x19839501; 30966854 PMC7168793

[35] Zhou Y, Zheng X, Xu B, Chen L, Wang Q, Deng H, et al. Circular RNA hsa_circ_0004015 regulates the proliferation, invasion, and TKI drug resistance of non-small cell lung cancer by miR-1183/PDPK1 signaling pathway. Biochem Biophys Res Commun. 2019;508(2):527–35. doi:10.1016/j.bbrc.2018.11.157; 30509491

[36] Lin S, Yu X, Yan H, Xu Y, Ma K, Wang X, et al. E2F7 serves as a potential prognostic biomarker for lung adenocarcinoma. Medicine. 2024;103(3):e34342. doi:10.1097/md.0000000000034342; 38241554 PMC10798722

[37] Hao F, Wang N, Zhang Y, Xu W, Chen Y, Fei X, et al. E2F7 enhances hepatocellular carcinoma growth by preserving the SP1/SOX4/Anillin axis via repressing miRNA-383-5p transcription. Mol Carcinog. 2022;61(11):975–88. doi:10.1002/mc.23454; 35924788 PMC9804269

[38] Xiang S, Wang Z, Ye Y, Zhang F, Li H, Yang Y, et al. E2F1 and E2F7 differentially regulate KPNA2 to promote the development of gallbladder cancer. Oncogene. 2019;38(8):1269–81. doi:10.1038/s41388-018-0494-7; 30254209

[39] Misetic H, Keddar MR, Jeannon JP, Ciccarelli FD. Mechanistic insights into the interactions between cancer drivers and the tumour immune microenvironment. Genome Med. 2023;15(1):40. doi:10.1101/2023.01.24.525325. 37277866 PMC10240791

[40] Yuan S, Almagro J, Fuchs E. Beyond genetics: driving cancer with the tumour microenvironment behind the wheel. Nat Rev Cancer. 2024;24(4):274–86. doi:10.1038/s41568-023-00660-9; 38347101 PMC11077468

[41] Wang Y, Pang J, Wang Q, Yan L, Wang L, Xing Z, et al. Delivering antisense oligonucleotides across the blood-brain barrier by tumor cell-derived small apoptotic bodies. Adv Sci. 2021;8(13):2004929. doi:10.1002/advs.202170079. PMC8261483 34258157

[42] Sun Y, Kong J, Ge X, Mao M, Yu H, Wang Y. An antisense oligonucleotide-loaded blood-brain barrier penetrable nanoparticle mediating recruitment of endogenous neural stem cells for the treatment of Parkinson’s disease. ACS Nano. 2023;17(5):4414–32. doi:10.1021/acsnano.2c09752; 36688425

[43] Barker SJ, Thayer MB, Kim C, Tatarakis D, Simon MJ, Dial R, et al. Targeting the transferrin receptor to transport antisense oligonucleotides across the mammalian blood-brain barrier. Sci Transl Med. 2024;16(760):eadi2245. doi:10.1126/scitranslmed.adi2245; 39141703

