# Supplementary material for: Competitive Sequestration of miR-1183 by lncRNA DDX11-AS1 Drives Gliomagenesis through E2F7 Activation
Source: Oncol Res. 2025 Sep 26;33(10):3023–40. doi: 10.32604/or.2025.065380 (PMC12494103; doi:10.32604/or.2025.065380)
Supplement: Supplementary file 1 [file OncolRes-33-65380-s001.docx]

**Table S1:** Clinical characteristics and prognosis of patients with glioma.

| **Patient No.** | **Sex** | **Age (Years)** | **WHO Grades** | **Histology** | **Primary or Recurrent** |
| --- | --- | --- | --- | --- | --- |
| 01 | M | 32 | 4 | GBM | Primary |
| 02 | M | 45 | 4 | GBM | Primary |
| 03 | M | 44 | 4 | GBM | Primary |
| 04 | F | 21 | 4 | GBM | Primary |
| 05 | M | 28 | 4 | GBM | Primary |
| 06 | M | 60 | 4 | GBM | Primary |
| 07 | F | 52 | 4 | GBM | Primary |
| 08 | M | 60 | 4 | GBM | Primary |
| 09 | M | 5 | 4 | GBM | Recurrent |
| 10 | M | 45 | 4 | GBM | Primary |
| 11 | M | 69 | 4 | GBM | Primary |
| 12 | F | 57 | 4 | GBM | Recurrent |
| 13 | M | 28 | 4 | GBM | Primary |
| 14 | M | 34 | 4 | GBM | Primary |
| 15 | F | 42 | 4 | GBM | Primary |
| 16 | M | 35 | 4 | GBM | Recurrent |
| 17 | F | 57 | 4 | GBM | Primary |
| 18 | M | 59 | 4 | GBM | Primary |
| 19 | F | 63 | 4 | GBM | Primary |
| 20 | M | 24 | 4 | GBM | Primary |
| 21 | M | 29 | 4 | GBM | Primary |
| 22 | F | 31 | 4 | GBM | Primary |
| 23 | M | 28 | 4 | GBM | Primary |
| 24 | F | 72 | 3 | LGG | Primary |
| 25 | M | 54 | 3 | LGG | Primary |
| 26 | M | 21 | 3 | LGG | Primary |
| 27 | M | 69 | 2 | LGG | Primary |
| 28 | M | 3 | 2 | LGG | Primary |
| 29 | M | 29 | 2 | LGG | Primary |
| 30 | F | 48 | 2 | LGG | Primary |
| 31 | M | 40 | 2 | LGG | Primary |
| 32 | F | 44 | 2 | LGG | Primary |

GBM: Glioblastoma; LGG: Low grade glioma; F: Female; M: Male; RT: Radiotherapy; CT: Chemotherapy.

**Table S2:** Primer sequences for RT-qPCR.

| **Gene Name** | **Sequences (5′ to 3′)** | |
| --- | --- | --- |
| DDX11-AS1 | Forward | CTGGGAAGCGTGCTTATTATGT |
|  | Reverse | AGGCTGACTGGAACAAGAGC |
| miR-1183 | Forward | ACTGACCACTGTAGGTGATGGT |
|  | Reverse | GCGAGCACAGAATTAATACGACTCACTATAGG |
| E2F7 | Forward | CAGAAACTGGAATCCCAACAAG |
|  | Reverse | TTAGTGGCTGGCTCATCCTCCTC |
| GAPDH | Forward | ATCAATGGAAATCCCATCACCA |
|  | Reverse | GACTCCACGACGTACTCAGCG |
| U6 | Forward | CTCGCTTCGGCAGCACATA |
|  | Reverse | CGAATTTGCGTGTCATCCT |
